# Epidemiological and clinical profile of fibromyalgia in Congolese patients at the university hospital of Kinshasa: a descriptive hospital-based study

**DOI:** 10.11604/pamj.2025.50.76.39291

**Published:** 2025-03-16

**Authors:** Aldo Nzita Mavinga, Jenny wa Mbuyi Mbuyi, Denis Tshitemb Matanda, Christophe Badibanga Mulumba, Aliocha Natuhoyila Nkodila, Pierrot Litite Lebughe, Jean-Marie Muamba Mbuyi, Jean-Jacques Kabasele Malemba

**Affiliations:** 1 Rheumatology Unit, Department of Internal Medicine, University Hospital of Kinshasa, Kinshasa, Democratic Republic of Congo,; 2 Family Medicine, Protestant University of Congo, Kinshasa, Democratic Republic of Congo

**Keywords:** Fibromyalgia, epidemiology, clinic, Kinshasa

## Abstract

Fibromyalgia is a controversial and often underreported clinical entity in routine medical practice. The present study aimed to describe its epidemiological and clinical profile in patients attending the University Hospital of Kinshasa. This descriptive hospital-based study was carried out in patients attending the rheumatology practice at the University Hospital of Kinshasa from December 2020 to March 2022. The following information was collected: age, sex, painful symptomatology, psychosomatic signs, the circumstances of the disease onset, factors that emphasize or reduce symptoms, the number of previous medical visits and the impact on socio-professional life. The diagnosis of fibromyalgia was defined according to the ACR 2010 criteria. Fibromyalgia was considered severe when it was associated with disability. Standard statistical tests were used to analyze the results. Five hundred and eighty-five (585) patients were selected during the study period. The diagnosis of fibromyalgia was retained in 63 of them, corresponding to a frequency of 10.8%. The sex ratio was 2 in favor of women and the average age was 50.9±12.4 years. The average diagnostic score was 17.6±3.6. Painful manifestations were dominated by arm involvement (84.1%). Fatigue was the most common psychosomatic manifestation (93.7%). Anxiety (41.3%) dominated the basic psychic state of patients and the average of previous medical visits was 5.2±1.6. Fibromyalgia was often triggered by emotional stress (44.4%). Quiet rest (42.9%) was the main calming factor. 60.3% of patients developed the severe form of the disease. Fibromyalgia concerns approximately one of ten patients who attend the rheumatology unit of the University Hospital of Kinshasa. It is more common in females and is associated with numerous psychosomatic signs in addition to the pain symptoms. Special attention must be paid to rheumatologists in order to ensure an adequate diagnostic approach.

## Introduction

Fibromyalgia remains a very controversial clinical entity. It presents as a painful, diffuse, chronic and disabling musculotendinous pathology, associated with fatigue and many collateral symptoms affecting various functions that are not well known [1,2]. Its prevalence in the general population is estimated between 1 to 2%, 90% of whom are women of 40 years old and above [3]. First described in 1977 by an author, Mease P, it was not until 1990 that the American College of Rheumatology (ACR) defined consensual classification criteria, and then in 1992 that the World Health Organization (WHO) included it as a rheumatic disease in the International Classification of Diseases [4].

Its main characteristic is a rich functional symptomatology contrasting with a clinical examination that does not contribute much, and paraclinical examinations that are often unremarkable [5]. However, it is very real for the people who suffer from it, often opening the door to a long period of medical wandering during which the patients can consult up to 10 doctors and more [6]. In addition, the lack of knowledge, the scepticism and sometimes the rejection of the medical profession led to complex situations, where we often observe an inflation of heavy assessments, unnecessary surgical interventions and inappropriate treatments [7]. All these factors listed above make the diagnosis of Fibromyalgia a real challenge for physicians and health professionals. Given all these parameters, it is very easy that the diagnosis, in this context, is either delayed, erroneous or wrongly directed towards psychiatry, clearly attesting to the frequent controversy associated with the diagnosis of Fibromyalgia [8].

Up to date, in the Democratic Republic of Congo, little attention has been paid to this pathology, so that there are no Congolese data on this disease. It is within this framework that the present study was carried out to describe the epidemiological and clinical profile of Fibromyalgia in rheumatology consultations at the University Hospital of Kinshasa.

## Methods

**Design, setting, and study population:** this is a descriptive hospital-based study of rheumatic patients seen between December 2020 and March 2022 in the rheumatology unit at the University Hospital of Kinshasa.

**Data collection:** all patients in this study were examined by a rheumatologist. Sociodemographic, anamnestic and physical examination data were recorded. The parameters of interest to define the clinical and epidemiological profile of fibromyalgia in the present study were: age, sex, location, frequency and nature of painful manifestations, psychosomatic signs, duration of symptoms, circumstances of onset, aggravating factors, lulling factors, medical-surgical history, number of previous medical visits, impact on socio-professional life and basic psychological state.

**Diagnostic criteria:** the diagnosis of fibromyalgia was based on the ACR 2010 criteria.

**Recruitment method, selection criteria, and bias:** patients were recruited consecutively. All new patients at least 18 years of age who freely consented to participate in the study and whose diffuse pain symptomatology had lasted at least 3 months were initially recruited. Of these recruited patients, those who met the criteria for diagnosis of fibromyalgia were included in the present study. Patients with diffuse pain syndrome in the context of sickle cell disease, endocrinopathy, functional colopathy or neoplastic pathology were excluded.

### Operational definitions

1) Fibromyalgia was considered severe when it was associated with disability, the latter being either a loss of autonomy or the inability to perform usual daily or professional tasks; 2) symptom regression was defined as a reduction of at least 30% of the initial pain symptomatology; 3) worsening of the clinical picture was defined as an increase of at least 30% of the initial pain symptomatology; 4) progression was considered stationary in situations intermediate between regression and aggravation; 5) the presentation of fibromyalgia pain attacks was considered intermittent when the attack lasted less than one day, discontinuous when the attack took several days in a row, and continuous when the attack could last at least 7 days.

**Statistical analysis:** statistical analysis was performed using the SPSS Statistics version 21.0. Descriptive analysis using numbers and percentages was used to present demographics features and the prevalence of Fibromyalgia. Continuous variables were described as mean ± standard deviation (SD). The student t-test was used to compare means. A nonparametric Chi-square test or Fisher's exact test was used to compare the different categorical variables between patients with and without severe fibromyalgia to look for factors associated with fibromyalgia severity. Subsequently, a p-value of less than 0.05 was considered statistically significant.

**Ethical considerations:** data were collected anonymously and confidentially after free and informed consent from each patient. The study adhered to the tenets of the Declaration of Helsinki and was approved by the ethics committee of the University of Kinshasa.

## Results

**Figure 1:**
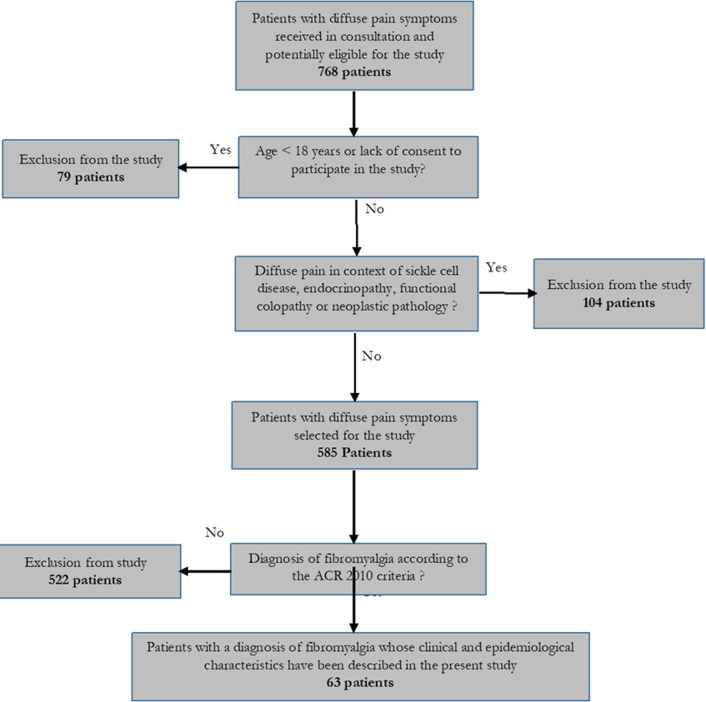
flow diagram showing number of patients at each stage of study

**General characteristics of the study population:** of a total of 585 patients received in rheumatology consultations and selected for the study (Figure 1), 63 met the criteria for fibromyalgia, corresponding to a relative frequency of 10.8%. The most affected population was women with a sex ratio of 2 in their favor. The average age of the patients was 50.9±12.4 years (Table 1).

**Table 1:** general characteristics of patients (n=63)

Parameters	Observations
**Sex**	
Male n (%)	21 (33.3)
Female n (%)	42 (66.7)
Average age (years)	50.9 ± 12.4
**Age range n (%)**	
< 50 years	31 (49.2)
50 - 59 years	15 (23.8)
≥ 60 years	17 (27.0)
Overall diagnostic score (WPI + SS)	17.6 ± 3.6
Painful areas (WPI)	11.6 ± 2.9
Duration of painful symptomatology (in months)	21.6 ± 11.6
Visual analog scale	6.2 ± 1.5
Severity of sympathetic signs (SS)	6.1 ± 1.7
Duration of sympathetic signs (in months)	45.3 ± 22.9
Physicians consulted	5.2 ± 1.6
**Medical history n (%)**	
Rheumatological history	63 (100.0)
Osteoarthritis	33 (52.4)
Chronic inflammatory rheumatism	19 (30.2)
Abarticular rheumatism	11 (17.5)
Digestive history	21 (33.3)
Depressive history	13 (20.6)
Urinary history	10 (15.9)

Categorical data are expressed as frequencies and percentages, while quantitative data are expressed as means and standard deviations; WPI: widespread pain index; SS: severity scale of sympathetic signs

**Figure 2:**
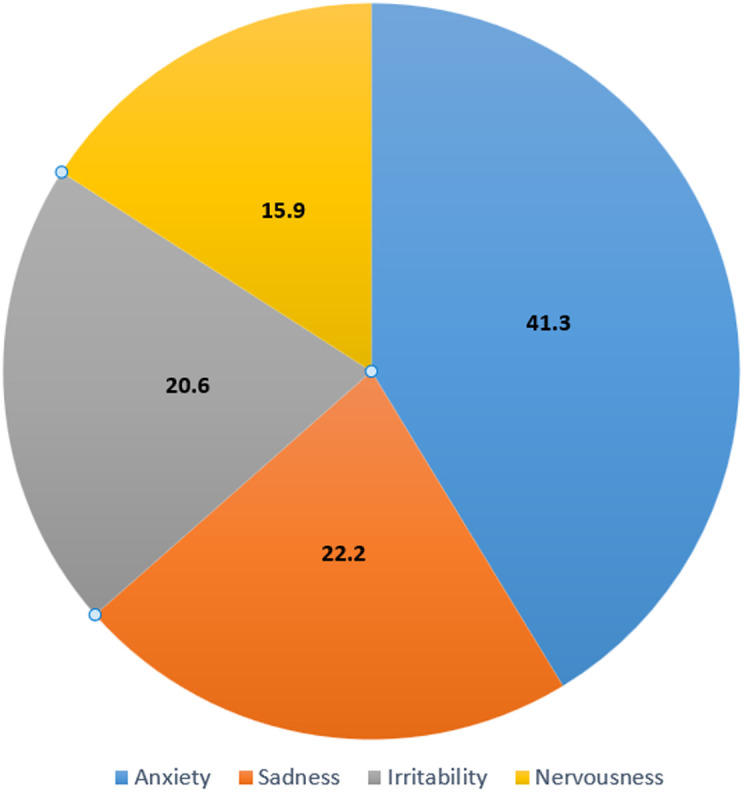
distribution of patients according to basic psychological state (n=63)

**Descriptive and analytic data:** fifty-point-eight percent (50.8%) of patients were at least 50 years old. The mean diagnostic score was 17.6±3.6 with 11.6±2.9 painful areas and 6.1±1.7 psychosomatic signs. The mean duration of painful symptoms was 21.6±11.6 months and that of psychosomatic signs 45.3±22.9 months. Patients had consulted an average of 5.2±1.6 physicians before arriving at Kinshasa University Hospital. Painful manifestations were dominated by the arms (84.1%), hips (69.8%), and legs (66.7%), and were of the gravity type in 44.4% of cases and presented as intermittent attacks in 47.6% of patients (Table 2). Fatigue was the most frequent psychosomatic manifestation, found in 93.7% of patients (Table 3). Emotional stress was the triggering factor in 44.4% of cases. Anxiety (41.3%) and sadness (22.2%) dominated the patients' basic psychological state (Figure 2). Aggravating factors were anxiety (34.9%) and cold (22.2%), whereas relief was provided by resting in a calm environment (42.9%) and practicing sports (23.8%) (Table 4). Severe fibromyalgia was observed in 60.3% of the patients, although no factor in the present study was associated with this severity (Table 5).

**Table 2:** frequency of algesic location, types and presentation of patients' pain (n=63)

Parameters	Observations n (%)
**Location**	
Neck	36 (57.1)
Temporo-mandibular	21 (33.3)
Shoulders	28 (44.4)
Arms	53 (84.1)
Forearms	39 (61.9)
Thorax	14 (22.2)
Abdomen	10 (15.8)
Back	28 (44.4)
Hips	44 (69.8)
Thighs	39 (61.9)
Legs	42 (66.7)
**Type**	
Gravity	28 (44.4)
Tingling	18 (28.6)
Crushing	10 (15.9)
Burning	7 (11.1)
**Presentation**	
Continuous	19 (30.2)
Intermittent	30 (47.6)
Discontinuous	14 (22.2)

**Table 3:** psychosomatic evaluation of patients (n=63)

Parameters	Observations n (%)
**Fatigue**	
No disorder	4 (6.3)
Mild disorder	13 (20.6)
Moderate disorder	28 (44.4)
Severe disorder	18 (28.6)
**Sleep disorders**	
No disorder	14 (22.2)
Mild disorder	25 (39.7)
Moderate disorder	18 (28.6)
Severe disorder	6 (9.5)
**Cognitive disorders**	
No disorder	27 (42.9)
Mild disorder	22 (34.9)
Moderate disorder	13 (20.6)
Severe disorder	1 (1.6)
**Somatic disorders**	
No disorder	21 (33.3)
Mild disorder	7 (11.1)
Moderate disorder	31 (49.2)
Severe disorder	4 (6.3)

**Table 4:** influencing factors, course and severity of disease (n=63)

Parameters	Observations n (%)
**Circumstance of onset n (%)**	
Emotional stress	28 (44.4)
Physical stress	13 (20.6)
Infectious episode	10 (15.9)
Not known	12 (19.0)
**Evolution n (%)**	
Worsening	25 (39.7)
Stationary	30 (47.6)
Regression	8 (12.7)
**Aggravating factors n (%)**	
Anxiety	22 (34.9)
Cold	14 (22.2)
Heat	8 (12.7)
Inactivity	11 (17.5)
Agitation	8 (12.7)
**Improving factors n (%)**	
Rest	27 (42.9)
Sport	15 (23.8)
Occupation	11 (17.5)
Entertainment	10 (15.9)
**Severity n (%)**	
Activity maintained	25 (39.7)
Disability	38 (60.3)

**Table 5:** factors associated with disease severity (n=63)

	Total; n=63	Activity maintained; n=25	Disability; n=38	P-value
Age (years)	50.9±12.4	51.3±12.7	50.7±12.4	0.854
Overall diagnostic score	17.6±3.6	17.2±4.0	17.9±3.4	0.508
WPI score	11.6±2.9	11.4±2.9	11.7±3.1	0.678
Painful duration (months)	21.6±11.6	21.4±12.2	21.7±11.4	0.915
Visual analog pain scale	6.2±1.5	6.3±1.6	6.1±1.4	0.600
Sympathetic signs score	6.1±1.7	5.9±1.9	6.2±1.6	0.502
Duration of sympathetic signs (months)	45.3±22.9	49.1±25.3	42.8±21.1	0.293
Physicians consulted	5.2±1.6	5.3±1.6	5.2±1.6	0.769

## Discussion

Fibromyalgia is a diffuse polyalgesic syndrome that has been evolving for at least 3 months and is associated with many other signs for which there is no unanimous pathophysiological hypothesis to date [9,10]. In the present study, fibromyalgia diagnosis corresponds to a relative frequency of 10.8%. The women were twice as affected as men with an average age of 50.9±12.4 years. The psychosomatic signs had been present for an average of 45.3±22.9 months and they often arrived before the painful manifestations. The frequency of 10.8% of fibromyalgia diagnosis in the present study seems to agree with the data in the literature suggesting a high prevalence of around 10-20% in rheumatology consultations [11]. This frequency is relatively higher than that found in general medical consultations (4 to 6%) and the general population (1 to 2%), as in France 1.4%, the USA 2%, Spain 2.4%, Italy 2.2%, Finland 0.75% and Denmark 0.66% [12]. These differences could probably be explained by the screening procedures, the diagnostic criteria used and the significant influence of secondary fibromyalgia cases in rheumatology consultations.

Women were twice as often affected as men, as has been shown in several studies throughout the world [13]. In the USA, fibromyalgia is 7 times more frequent in women (3.4%) than in men (0.5%), in Spain it is 20 times more frequent in women (4.2%) than in men (0.2%) and in Finland it is twice as important in women (0.98%) as in men (0.48%). This preponderance of women seems to be the result of several factors involving a supposed female susceptibility to pain, different psychological and socio-cultural experiences of pain, but also mechanisms of amplification and neurological inhibition of pain with a genetic or hormonal support [13,14].

The average age of the study population was 50.9±12.4 years, results similar to the series of Girard in Switzerland [15], Aïni *et al*. [16] in France and Guich *et al*. in Morocco [17] with mean ages of 50±9.8, 50.4±6.9 and 57±11.5 years respectively. Fifty-point two percent (50.2%) of the fibromyalgia patients were older than 50 years in the present study. In France, Jasson [18] found 65.7% of patients to be over 50 years of age. These data are in agreement with the literature which describes that fibromyalgia frequently appears in early or middle adulthood and its incidence tends to increase with age.

The diagnosis of fibromyalgia in the present study was made based on the ACR 2010 criteria with an average diagnostic score of 17.3±3.6. This score includes a mean of 11.6±2.9 for painful areas and 6.1±1.7 for psychosomatic signs. The psychosomatic signs, including almost constant fatigue (93.7%), had been present for an average of 45.3±22.9 months. They often arrived before the painful manifestations (24±11.6 months), and led the patients to a long period of medical wandering with 5.2±1.6 doctors consulted. These results are close to the series of Jasson [18] in France who found an average of 6 doctors consulted. It appears that in the majority of fibromyalgia patients, psychosomatic signs are present well before the onset of the diffuse polyalgesic syndrome characterizing the state period of the disease. To allow an early diagnosis, it would be necessary to institute a systematic search for these warning signs in all rheumatic patients with polyalgia who are refractory to the usual treatments [19-21].

Emotional stress was the triggering factor for fibromyalgia in 44.4% of cases, and its clinical manifestations were regressive in only 12.7% of cases. The basic psychological state of the patients was dominated by anxiety (41.3%). The aggravating factor was anxiety in 34.9% of the cases and the predominant factor of improvement was resting in calm (42.9%). Guich *et al*. [17] in Morocco reported a feeling of injustice in 41.7% of the fibromyalgia patients in their series, whereas Alagnide *et al*. [22] in Benin found the anxiety-depression syndrome to be the main etiological factor in 95% of the patients. All these data prove that there is a very important psychological component in this pathology, which sometimes explains the failure of isolated drug therapies. It is therefore necessary to have a multidisciplinary approach to the patients concerned, including psychological, medico-social and physical care [23-27].

Disability as the ultimate consequence of the negative evolution of fibromyalgia was found in 60.3% of cases. Jasson [18] in France and Alagnide *et al*. [22] in Benin found a clear disruption of socio-professional life in 63% and 80% of patients. This disability is associated with fibromyalgia and is frequently reported in several studies and the literature. It is often correlated with the importance of the painful areas and the duration of the symptoms [28-30]. In the present study, no factor was associated with the severity of the disease. This data should be considered with caution given the small sample size.

This descriptive hospital-based study is the very first study which reports the frequency of fibromyalgia in the Congolese environment, its clinical characteristics and impact on rheumatic patients. Particular attention should be paid to it because fibromyalgia remains an under-diagnosed pathology in daily practice [31-33]. The present study showed a significant frequency of the disease using a current diagnostic tool with high sensitivity and specificity. However, the small sample size remains the major factor that can limit the extrapolation of the results of the present work, which requires large-scale studies for their confirmation.

## Conclusion

Fibromyalgia is a condition frequently encountered in the rheumatology practice of the University Hospital of Kinshasa. It affects approximately one patient out of ten. It is mostly female and is associated with numerous psychosomatic signs in addition to the painful symptoms. Its diagnosis is often delayed because of this broad and complex clinical symptomatology, initially dominated by psychosomatic signs. Special attention must be paid to rheumatologists to ensure an adequate diagnostic approach.

### What is known about this topic

- Fibromyalgia remains to this day a controversial and often under-reported clinical entity, despite its recognition by the World Health Organization since 1992;
- Western countries are the most affected in the world, while in sub-Saharan Africa, it is thought to be rare, like most poorly understood rheumatic diseases;
- Some factors seem to be very involved in the onset of fibromyalgia such as psychological trauma, physical effort, overwork or a serious infectious episode.

### What this study adds

- This is the first study in the Democratic Republic of Congo to describe the epidemiological and clinical profile of fibromyalgia;
- This study showed that fibromyalgia is a frequent reason for consultation in rheumatology among Congolese patients;
- It also showed the important part of psychosomatic signs in the often late diagnosis of fibromyalgia in Congolese patients.
